# Advances in Stem Cell Therapies for Rotator Cuff Injuries

**DOI:** 10.3389/fbioe.2022.866195

**Published:** 2022-05-25

**Authors:** Hao-Nan Wang, Xiao Rong, Lu-Ming Yang, Wei-Zhong Hua, Guo-Xin Ni

**Affiliations:** ^1^ School of Sport Medicine and Rehabilitation, Beijing Sport University, Beijing, China; ^2^ Department of Ultrasound, West China Hospital, Sichuan University, Chengdu, China; ^3^ Musculoskeletal Sonography and Occupational Performance Lab, Chan Division of Occupational Science and Occupational Therapy, University of Southern California, Los Angeles, CA, United States

**Keywords:** rotator cuff, stem cell, extracellular vesicle, exosome, biologic, regenerative medicine

## Abstract

Rotator cuff injury is a common upper extremity musculoskeletal disease that may lead to persistent pain and functional impairment. Despite the clinical outcomes of the surgical procedures being satisfactory, the repair of the rotator cuff remains problematic, such as through failure of healing, adhesion formation, and fatty infiltration. Stem cells have high proliferation, strong paracrine action, and multiple differentiation potential, which promote tendon remodeling and fibrocartilage formation and increase biomechanical strength. Additionally, stem cell-derived extracellular vesicles (EVs) can increase collagen synthesis and inhibit inflammation and adhesion formation by carrying regulatory proteins and microRNAs. Therefore, stem cell-based therapy is a promising therapeutic strategy that has great potential for rotator cuff healing. In this review, we summarize the advances of stem cells and stem cell-derived EVs in rotator cuff repair and highlight the underlying mechanism of stem cells and stem cell-derived EVs and biomaterial delivery systems. Future studies need to explore stem cell therapy in combination with cellular factors, gene therapy, and novel biomaterial delivery systems.

## 1 Introduction

Rotator cuff injury is one of the leading musculoskeletal diseases worldwide and the most common condition that leads to the complaint of shoulder pain (Picavet and Schouten, 2003). It is estimated that the prevalence of shoulder problems in primary care is 2.4% in the UK (Linsell et al., 2006), and 30%–70% of shoulder pain results from rotator cuff diseases (Mitchell et al., 2005). Intrinsic factors contribute to rotator cuff disease, including age, obesity, smoking, diabetes mellitus, genetics, and narrow anatomical subacromial spaces (Titchener et al., 2014). Among these factors, age-related degeneration is considered the main reason for rotator cuff disease, and the prevalence of rotator cuff tears increases with age in the general population. It was found that the prevalence of sonographic full-thickness rotator cuff tears was 10.7% in the 50s, 15.2% in the 60s, 26.5% in the 70s, and 36.6% in the 80s (Minagawa et al., 2013). While the intrinsic risks decrease the structural resilience of rotator cuff, the extrinsic risks, such as occupations and sports activities, cause excessive mechanical loading on it, involving rotator cuff injury (Whittle and Buchbinder, 2015). According to the continuum of the tendon pathology model, mechanical loading plays an important role in pathological changes (Lewis, 2010), and a repeated and biomechanical loading on the rotator cuff tendon increases the risk of rotator cuff injury (Edmonds and Dengerink, 2014). Rotator cuff injuries may start from tendinopathy and progressively develop into partial or complete tendon tears (Lewis, 2010), which typically result in pain, loss of motion, and functional impairment of the shoulder (Craig et al., 2017). Generally, tendinopathy does not cause substantial problems; therefore, patients with tendinopathy are initially recommended for a course of conservative management, such as physiotherapy and analgesia (Seida et al., 2010). In some cases, patients with tendinopathy may have an increased risk of tendon rupture, especially among those in the older population (Yasui et al., 2017). Nevertheless, acute shoulder trauma may cause partial or complete tendon tears, which require surgical treatment to repair the continuity of the structure or surgery to reattach the tendon back to its bony insertion.

A previous systematic review that included 15 studies and 371 patients after rotator cuff injury demonstrated improved clinical outcomes with an earlier time of receiving surgery (Mukovozov et al., 2013). Unfortunately, the clinical outcomes remained after surgery, and the overall failure rate of healing was 43% at 12 months postsurgical repair (Rashid et al., 2017), and even up to 90% in the elderly (Galatz et al., 2004). The rehabilitation process following rotator cuff arthroscopic repair usually lasts for a few months, and athletes take over 6 months to return to sports (Thigpen et al., 2016). Additionally, the formation of scar tissue at the injury site can cause tissue adhesion and joint stiffness, as well as poor mechanical properties, which increase the risk of retear (Thomopoulos et al., 2010). Due to these issues, there has been a growing interest in the past decade in preparing stem cells to enhance rotator cuff repair and regeneration. Mesenchymal stem cells (MSCs) are the most popular stem cells because of their accessibility to multiple tissues, anti-inflammatory properties, secretion of trophic factors, and differentiation ability into tenocytes to recellularize the regenerating tissue (Lim et al., 2019). In this review, we summarize the advances of stem cells and stem cell-derived extracellular vesicles in rotator cuff repair, gene therapy, and their biomaterial delivery systems.

## 2 Rotator Cuff Structure and Healing

The rotator cuff comprises four muscles, namely, supraspinatus, infraspinatus, subscapularis, and teres minor muscles, which envelop the shoulder joint and attach closely to the humeral head *via* their tendons (Escamilla and Andrews, 2009; Oliva et al., 2015). These muscles play a critical role in both movement and dynamic stabilization during the locomotion of the shoulder joint (Lin et al., 2018). The particular anatomy of rotator cuff and lack of blood vessels can lead to injuries that cannot be healed easily or effectively (Hegedus et al., 2010). Rotator cuff injury often involves the entire muscle–tendon–bone complex, of which the tendon and tendon–bone interface are the most frequently injured and concerned sections.

The tendon is a unique form of connective tissue that transmits muscle-contraction force to the skeleton to maintain posture or produce motion. It comprises resident cells and the extracellular matrix (ECM). Tenocytes, the main type of cell located inside collagen fibers, produce collagen I and ECM molecules (Nourissat et al., 2015). There are tendon stem/progenitor cells (TSPCs), also commonly termed tendon-derived stem cells (TDSCs), which are capable of renewing tenocytes through differentiation and proliferation to maintain homeostasis (Bi et al., 2007). The ECM contains multitudinous molecules, including collagen, elastin, proteoglycans, and glycoproteins, which are involved in tendon-specific collagen I. The triple-helical collagen I molecules are assembled into fibrils that, in turn, form fibers, fascicles, and, ultimately, tendons. Of these, the collagen fibrils are considered to be the basis for force transmission (Kannus, 2000).

**FIGURE 1:**
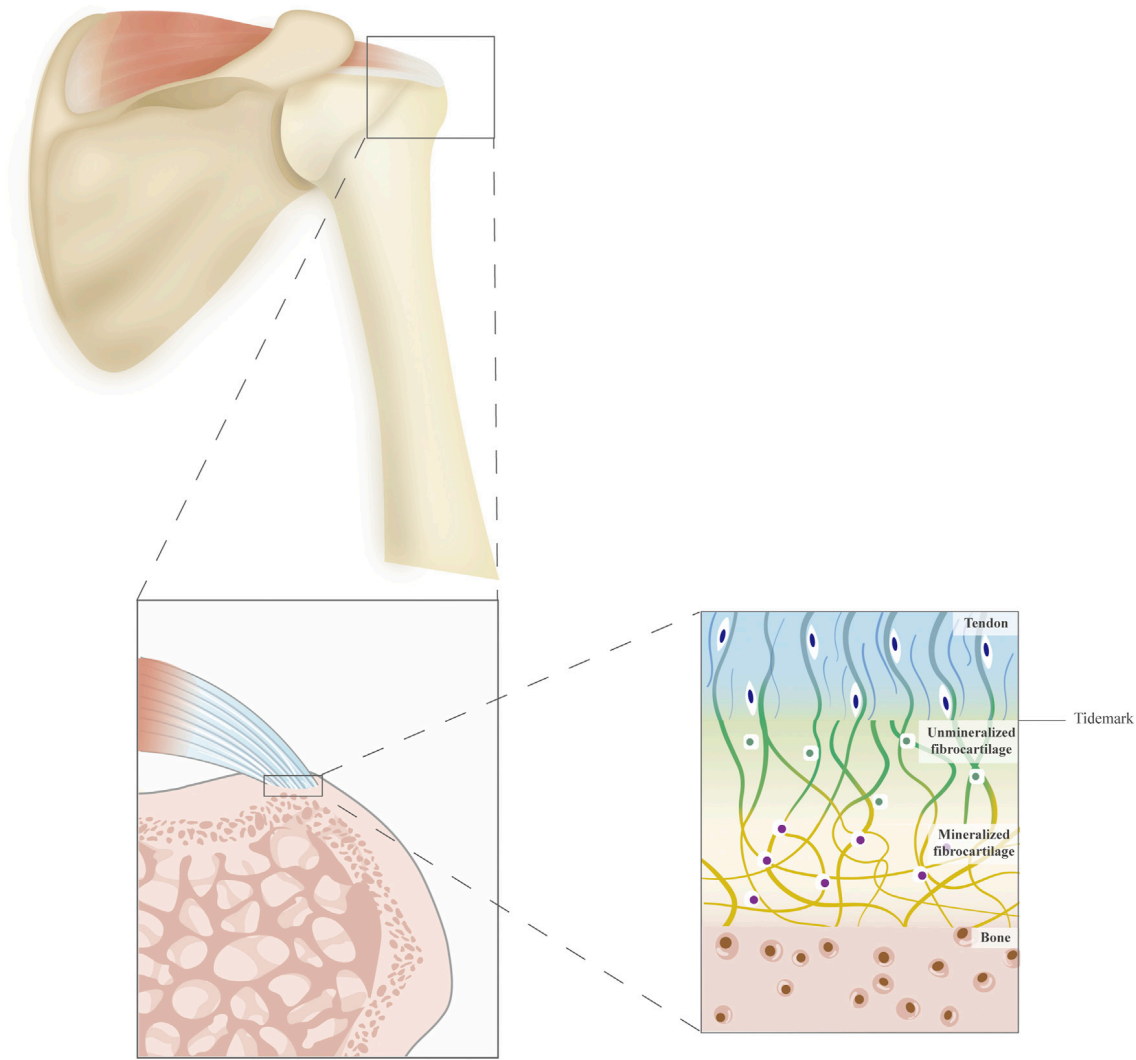
A schematic diagram of the supraspinatus tendon and the structure of the tendon–bone interface. The tendon–bone interface is divided into four continuous but distinct zones: tendon, unmineralized fibrocartilage, mineralized fibrocartilage, and bone.

The tendon–bone unit is a specialized structure called an enthesis, which represents a transition between soft tendinous and hard bony tissue (Yang and Temenoff, 2009; Andarawis-Puri et al., 2015) (Figure 1). Various resident specialized cell types are found in this tissue, including osteoblasts, osteocytes, osteoclasts, fibrochondrocytes, and tenocytes. The enthesis has been divided into four continuous but distinct zones: tendon, non-mineralized fibrocartilage, mineralized fibrocartilage, and bone (Thomopoulos et al., 2010). Zone 1 (tendon area) mainly consists of collagen I fibers together with a small amount of decorin. Zone 2 (non-mineralized fibrocartilage) predominantly contains collagen II and III fibers, as well as small amounts of collagen I, IX, and X–collagen fibers. The decorin and aggregates also exist. In Zone 3 (mineralized fibrocartilage), the fibrocartilage is mineralized and inserted into the subchondral bone layer. Both aggrecan and mineral components are present in the extracellular matrix composition. Zone 4 (bone area) is mainly a bone-like composition that contains collagen I fiber mineralized in osteoblasts, osteocytes, and osteoclasts. The gradual changes in microstructure allow for mechanical strain, stress distribution, and efficient energy transition (Rossetti et al., 2017; Takahashi et al., 2017).

Following a rotator cuff tear, the injured site undergoes a natural healing process involving three overlapping stages—inflammatory, proliferation, and remodeling (Docheva et al., 2015). In the inflammatory stage, inflammatory cells are attracted to the injury site by pro-inflammatory cytokines, such as neutrophils, monocytes, and macrophages and they yield inflammatory cytokines, including interleukin (IL)-6 and IL-1β (Lin et al., 2004). Additionally, various growth factors are released by cells to promote tissue repair, such as basic fibroblast growth factor (bFGF), bone morphogenetic proteins (BMPs), transforming growth factor-beta (TGF-β), and vascular endothelial growth factor (VEGF) (Docheva et al., 2015). The angiogenic factors induce the formation of a neovascular network that handles the blood supply for newly formed fibrous tissue (Fenwick et al., 2002; Hegedus et al., 2010). Meanwhile, the tenocytes are recruited to the wounded site and induced to proliferate. The second stage is characterized by the abundant synthetic activity of the ECM component; predominately collagen III, directed by recruited fibroblasts, which leads to disorganized alignment of the tendon and mechanical weakness. In the remodeling stage, the density of cells and the synthesis of ECM components both decrease. At the same time, collagen III is gradually replaced by collagen I, which induces the ECM of the tendon to become more aligned; meanwhile, tendon stiffness and tensile strength are restored to the pre-injury level (Voleti et al., 2012).

The healing process involves both intrinsic and extrinsic healing processes (Longo et al., 2011). The extrinsic healing process initially occurs, which involves the invasion of extrinsic cells from the surrounding sheath and synovium, resulting in scar tissue formation to substitute for the native enthesis. The formation of scar tissue and the absence of fibrocartilage lead to the secretion of collagen III fibers rather than collagen I fibers. Then, intrinsic healing is activated and simultaneously induces the tenocyte recruitment, proliferation, and secretion of collagen I fibers, which can strengthen the mechanical property of the tendon (Docheva et al., 2015). The scar tissue lacks the gradient of mineral distribution, and the diameter of collagen III fibers is smaller than that of collagen I fibers (Hexter et al., 2017). For this reason, the structure will exhibit weak mechanical properties. Although collagen III fibers can be replaced by collagen I fibers, it usually takes up to 12 months to complete the healing process, which may lead to a higher chance of re-tearing (Lee et al., 2019; Haleem et al., 2021).

## 3 Stem Cells for Rotator Cuff Injury

### 3.1 Stem Cell-Based Therapy

**FIGURE 2:**
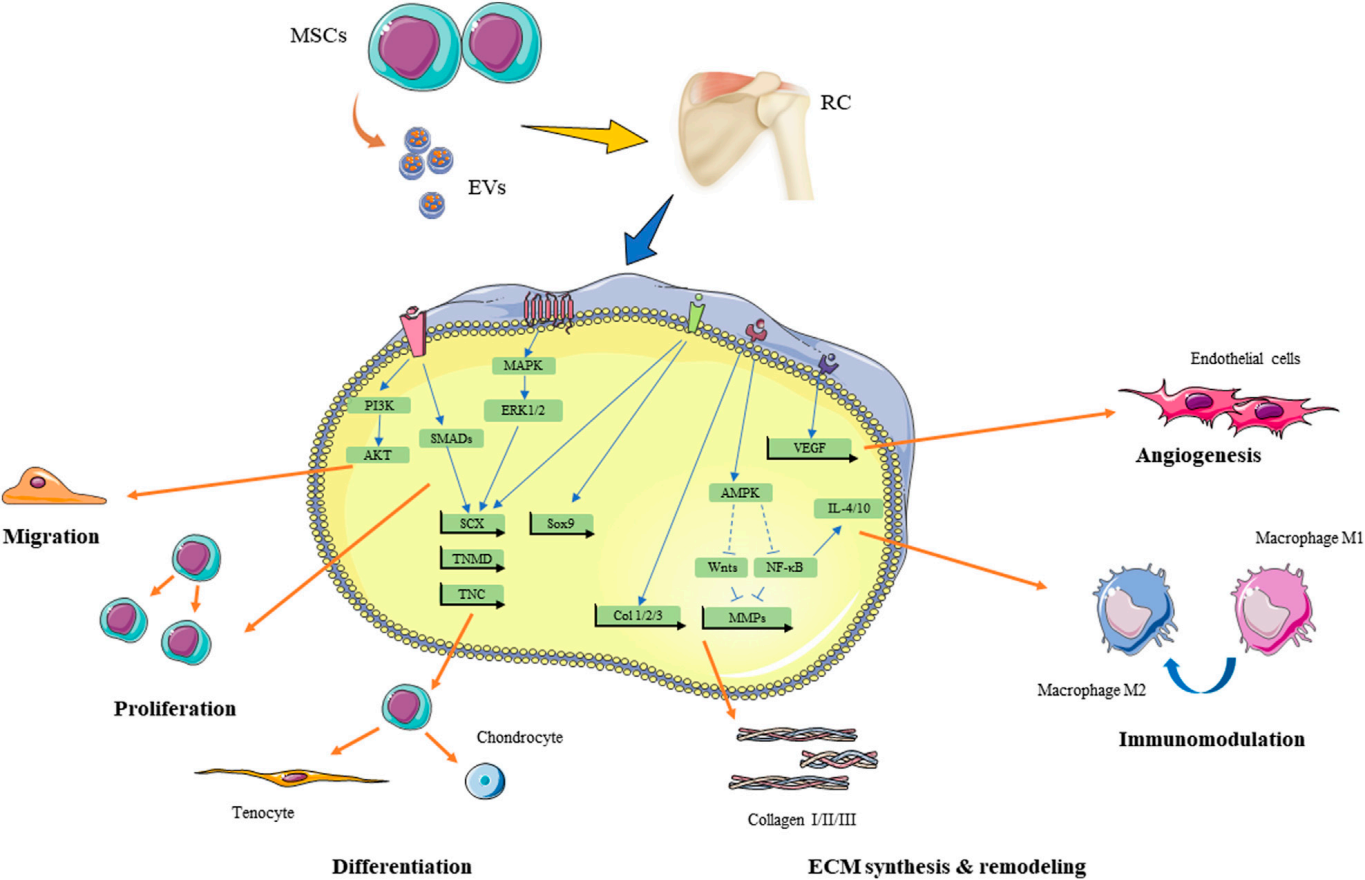
Theraputic effect of MSCs and MSC-EVs for rotator cuff injuries and underlying signaling pathways. RC, rotator cuff; MSCs, mesenchymal stem cells; EVs, extracellular vesicles; MAKP, mitogen-activated protein kinase; ERK, extracellular signal-regulated kinases; PI3K, phosphoinositide 3-kinase; SCX, scleraxis; TNMD, tenomodulin; TNC, tenascin C, Sox9, SRY-Box transcription factor 9; Runx2, runt-related transcription factor 2; AMPK, 5′ AMP-activated protein kinase; NF-κB, nuclear factor kappa B; MMPs,matrix metalloproteinases; IL, interleukin. ECM, extracellular matrix.

The use of MSCs is a promising and outstanding therapeutic efficacy in regenerative medicine. The stem cells have been successfully derived from multiple tissues, including bone marrow, adipose tissue, tendon, umbilical cord blood, bursa, and urine. In this review, we summarize the animal and clinical studies of these stem cells for rotator cuff injuries. The therapeutic effects of MSCs for rotator cuff injuries are summarized in Figure 2.

#### 3.1.1 Bone Marrow-Derived Mesenchymal Stem Cells

Bone marrow-derived mesenchymal stem cells (BMSCs) are the first-discovered mesenchymal stem cells, which act as pluripotent cells (Heo et al., 2016) with multilineage differentiation ability (Docheva et al., 2007; Dai et al., 2015; Perucca Orfei et al., 2019) into adipocytes, osteoblasts and chondrocytes, and tenocytes. Therefore, they have been used in various tissue repairs and regeneration procedures. The principal source of BMSCs in rotator cuff injury is autologous cells that can be harvested from the iliac crest and proximal humerus.

Kida et al. (2013) found that drilled holes in the humerus footprint could stimulate autologous BMSCs to infiltrate into the repair site to promote tendon–bone healing by enhancing the ultimate force-to-failure. Exogenous BMSC can be delivered to the repair site by various carriers (Chen P. et al., 2020). The untreated BMSCs increased the early formation of fibrocartilage and collagen orientation as well as biomechanical strength at 2 weeks. The enhancement of fibrocartilage formation is due to the higher chondrogenesis expression, such as *SRY-Box Transcription Factor 9* (*Sox9*), *COL2A1*, and *aggrecan*, during tendon–bone healing (Alves de Araújo et al., 2012). However, it seemed that the effect augmented with BMSCs dissipated by 4 weeks (Degen et al., 2016). In contrast, Gulotta et al. (2009) found no differences amounting to new cartilage formation, collagen fiber organization, or biomechanical strength at either two or four weeks. Additionally, bioactive factor-induced BMSCs could achieve better efficiency in promoting tissue regeneration than BMSCs alone. For instance, BMSCs endowed with platelet-rich plasma (PRP) enhanced the production of growth factors, the ability of osteogenic differentiation, and the resistance of cell death *in vitro*, and they promoted bone formation and the biomechanical property of the newly generated bone *in vivo* (Han et al., 2019). The therapeutic effects of BMSCs for rotator cuff injuries are summarized in Table 1.

**TABLE 1:** Summary of bone marrow mesenchymal stem cells for rotator cuff injuries.

	Animal	Injury model	Type of cells	Method of delivery	Time of observation	Results
Gulotta et al., (2009)	Rat	Acute tear and repair of supraspinatus tendon	BMSC	Fibrin glue carrier	2 and 4 weeks	No improvement on the structure, composition, or strength of the healing tendon attachment
Kida et al., (2013)	Rat	Acute tear and repair of supraspinatus tendon	BMSC	Transosseous drilling	2, 4, and 8 weeks	BMSC infiltrated the repaired tendon, and improved biomechanical property
Degen et al., (2016)	Rat	Acute tear and repair of supraspinatus tendon	BMSC	Fibrin glue carrier	2 and 4 weeks	Improved early histologic appearance and biomechanical strength
Thangarajah et al., (2018)	Rat	Chronic tear and repair of supraspinatus tendon	BMSC	A demineralized bone matrix with Fibrin glue carrier	6 weeks	Enhanced rotator cuff healing and restore bone mineral density at the enthesis
Liu et al., (2019)	Dog	Acute tear and repair of infraspinatus tendon	BMSC	Engineered tendon–fibrocartilage–bone composite with BMSC cell sheet	6 weeks	Enhanced rotator cuff anatomic structure, collagen organization and biomechanical strength
Han et al., (2019)	Rat	Acute tear and repair of supraspinatus tendon	BMSC	Not mentioned	4 and 8 weeks	Enhanced biomechanical property of the newly generated bone
Chen P. et al., (2020)	Rabbit	Acute tear and repair of supraspinatus tendon	BMSC	3D-printed PLGA Scaffolds	4, 8 and 12 weeks	Enhanced collagen formation and increased collagen diameter in the tendon–bone interface, and improved the biomechanical properties
Gulotta et al., (2010)	Rat	Acute tear and repair of supraspinatus tendon	BMSC transfected with MT1-MMP	Fibrin glue carrier	2 and 4 weeks	Presence of more fibrocartilage at the insertion and improved biomechanical strength
Gulotta et al., (2011b)	Rat	Acute tear and repair of supraspinatus tendon	BMSC transfected with BMP-13	Fibrin glue carrier	2 and 4 weeks	No differences in the histologic appearance and biomechanical strength of the repairs
Gulotta et al., (2011a)	Rat	Acute tear and repair of supraspinatus tendon	BMSC transfected with sleraxis	Fibrin glue carrier	4 and 8 weeks	Improved histologic appearance with more fibrocartilage and higher biomechanical strength

Some clinical studies have investigated the effectiveness of BMSCs for patients with arthroscopic rotator cuff repair. The procedure of multiple channeling for rotator cuff repair creates holes in the greater tuberosity to promote endogenous BMSCs of the proximal humerus infiltrating into the repair site. A cohort study found no difference between groups that underwent arthroscopic rotator cuff repair with multiple channeling and those without channeling in clinical and structural outcomes at a follow-up of 2 years (Jo et al., 2013). However, another study suggested that patients who accepted the multiple channeling procedures reported a significantly lower retear rate, indicating that BMSCs may improve structural integrity for arthroscopic rotator cuff repair (Hernigou et al., 2014). Taniguchi et al. (2015) reported that applying bone marrow stimulation to the footprint during arthroscopic surface-holding (ASH) repair resulted in improved cuff repair integrity based on Sugaya’s classification by postoperative magnetic resonance imaging, particularly in large-massive tears. In a case-control study, the healing rates of BMSC augmented repair and repair only during arthroscopy were 100% and 67%, respectively. Furthermore, the augmentation of BMSCs prevents further tears after a follow-up of 10 years (Hernigou et al., 2014).

#### 3.1.2 Adipose-Derived Stem Cells

In recent years, it has been attractive to use adipose-derived stem cells (ADSCs) to enhance rotator cuff repair because of their easy acquisition and ability to inhibit osteogenic differentiation by modulating the microenvironment and anti-inflammatory properties (Bunnell et al., 2008; Kokubu et al., 2020). ADSCs are an ideal source of stem cells in regeneration therapy due to their accessibility; they can be isolated in large quantities from subcutaneous adipose tissue (Bunnell et al., 2008) and liposuction aspirates (De Francesco et al., 2015). As undifferentiated stem cells, they have high proliferation rates and potentially differentiate into tenocytes with growth factors and mechanical stress (Dai et al., 2015; Rinella et al., 2018).

In addition, ADSCs have shown similar therapeutic effects to BMSCs in rotator cuff regeneration. It showed that ADSCs mediated acute inflammation with diminished presence of edema and neutrophils but did not improve the biomechanical properties of tendon–bone healing from two to eight weeks after repair in a rat acute rotator cuff repair model (Mora et al., 2014; Barco et al., 2015). Lipner et al. (2015) reported that ADSCs could reverse the dominated fibrovascular scar response in acute tendon–bone healing. Compared to acute rotator cuff injury, chronic rotator cuff injury causes bone loss and reduced structural properties. ADSCs transplanted to the injured site can increase the bone mineral density of the proximal humerus to promote tendon–bone healing in repairs of chronic tears (Kaizawa et al., 2019; Rothrauff et al., 2019; Shin et al., 2020). Additionally, injection of ADSCs into the musculotendinous junction area of the subscapularis can improve muscle function by electromyographic evaluation and decrease fatty infiltration of the muscle, and it tends to enhance the load-to-failure in chronic rotator cuff tears (Oh et al., 2014). The therapeutic effects of ADSCs for rotator cuff injuries are summarized in Table 2.

**TABLE 2:** Summary of adipose stem cells for rotator cuff injuries.

	Animal	Injury model	Type of cells	Method of delivery	Time of observation	Results
Oh et al., (2014)	Rabbit	Chronic tear and repair of subscapularis tendon	ADSC	Balanced salt solution	6 and 12 weeks	Improved muscle function and decreased fatty infiltration, but no significant different biomechanical strength after cuff repair
Mora et al., (2014)	Rat	Acute tear and repair of supraspinatus tendon	ADSC	Collagen carrier	2 and 4 weeks	Less inflammation, but no improvement of biomechanical property
Barco et al., (2015)	Rat	Acute tear and repair of supraspinatus tendon	ADSC	Fibrin sealant	4 and 8 weeks	Less presence of neutrophils and more presence of plasma cells without improving histologic appearance and biomechanical strength
Lipner et al., (2015)	Rat	Acute tear and repair of supraspinatus tendon	ADSC/ ADSC transfected with BMP-12	PLGA nanofibers with gradients in mineral with fibrin hydrogel	2, 4 and 8 weeks	ADSC transfected with BMP-12 decrease mechanical properties, strength, and modulus in the repair site
Rothrauff et al., (2019)	Rat	Acute tear and repair of the supraspinatus and infraspinatus Tendons/ Chronic Intramuscular injection of botulinum toxin A and repair	ADSC	GelMA/fibrin hydrogel	4 weeks	Higher bone mineral density of the proximal humerus in chronic model with both GelMA/Fibrin hydrogel delivery system
Kaizawa et al., (2019)	Rat	Chronic tear and repair of supraspinatus tendon	ADSC	Human tendon hydrogel	8 weeks	Tendon hydrogel augmentation with ADSC improves biomechanical properties and fibrocartilage area than no treatment, but no improvement of tendon–bone interface than tendon hydrogel alone
Shin et al., (2020)	Rat	Chronic tear and repair of supraspinatus tendon	ADSC	Engineered cell sheets	2 and 4 weeks	Larger fibrocartilage area, higher bone volume/total volume values, and biomechanical property

ADSCs are some of the most commonly used stem cells in clinical research on rotator cuff injuries. The cohort study by Kim et al. (2017) revealed that 182 patients treated with an injection of ADSCs loaded in fibrin glue (4.46 × 10^6^ cells) during arthroscopic rotator cuff repair could significantly improve structural outcomes assessed in terms of the retear rate, and MRI results indicated that the retear rate of the ADSC group was less than that of the control group (14.3% and 28.5%, respectively) at a minimum of 12 months after surgery. However, there were no significant differences in pain intensity, range of motion, or self-reported function at 28 months of follow-up.

For patients with symptomatic partial-thickness rotator cuff tears (sPTRCT), surgery may not be the first-choice therapy; thus, many studies have used ADSC therapy as a regeneration strategy. In a pilot RCT study, patients with sPTRCT who did not respond to physical therapy treatments for at least 6 weeks were randomly assigned to receive a single injection of unmodified, autologous adipose-derived regenerative cells (UA-ADRC) (11.4 × 10^6^ cells) or a single injection of 80 mg of methylprednisolone (40 mg/ml; 2 ml) plus 3 ml of 0.25% bupivacaine. The patients in the UA-ADRC group showed significantly higher total scores on the American Shoulder and Elbow Surgeons Standardized Shoulder Assessment Form (ASES) at 3 and 12 months post-treatment compared to those in the corticosteroid group. No severe adverse events related to the injection of UA-ADRCs were reported at the 12-month post-treatment follow-up (Hurd et al., 2020). A retrospective comparative study showed that a high-dose (1.0 × 10^8^ cells) intratendinous injection of ADSCs for patients with sPTRCT can improve shoulder function scores and rotator cuff strength for up to 2 years post-treatment. The results of the MRI showed that the bursal-sided defects nearly disappeared at 1 year and did not recur for up to 2 years. Importantly, there were no treatment-related adverse events at a minimum 2-year follow-up (Jo et al., 2020).

#### 3.1.3 Tendon Stem/Progenitor Cells

Progenitor/tendon stem cells (TSPCs), also known as tendon-derived stem cells (TDSCs), were isolated and identified from humans and mice in 2007 (Bi et al., 2007). TSPCs are so named because they can be harvested and isolated from the tendon of the supraspinatus and the long head of the biceps during arthroscopic rotator cuff repair procedures (Tsai et al., 2013; Dei Giudici and Castricini, 2020). Like other MSCs, TPSCs have characteristics of high clonogenicity, self-renewal capacity (Al-Ani et al., 2015), and multi-differentiation potential, including tenocytes, chondrocytes, osteocytes, and adipocytes (Zhang and Wang, 2010; Leonardi et al., 2021). They have highly expressed tendon-related genes, including *COL1A1*, *tenascin C* (*TNC*), *Scleraxis* (*Scx*), and *Tenomodulin* (*TNMD*), which may contribute to spontaneous tenogenic differentiation (Guo et al., 2016). Therefore, TPSCs are a promising source of tendon regeneration. However, owing to their abundance in the tendon, it is challenging to obtain autologous TSPCs, which could limit their application in clinical studies.

Several studies have investigated the utilization of TPSCs to treat tendon disorders in pre-clinical studies (Song et al., 2018). Shen et al. (2012) used allogenic TSPC-seeded scaffolds to augment rotator cuff repair in a rabbit model. There was no elicited immune response, with decreasing lymphocytic infiltration at early repair and improving histological and biomechanical properties compared to non-TSPC treatment control repairs at 12 weeks post-surgery. Furthermore, it showed that transplanting the cell sheet that was derived from the rotator cuff promoted cartilage regeneration and angiogenesis at the enthesis and upregulated the expression of genes *VEGF* and *COL2A1* at 4 weeks and a greater ultimate failure load at 8 weeks after surgery (Harada et al., 2017).

#### 3.1.4 Umbilical Cord-Derived Mesenchymal Stem Cells

Umbilical cord-derived mesenchymal stem cells (UCB-MSCs) are a promising source of human cells because of their easy availability, high proliferation capacity, and low immunogenicity (Wang et al., 2009; Bai et al., 2016). The advantage of UCB-MSCs is that allogeneic stem cells do not require autologous tissues, such as bone marrow aspiration and adipose tissue (Kasper et al., 2009). Therefore, UCB-MSCs can be prepared early before treatment, and the function of stem cells is not affected by the age of patients or disease. Park et al. (2015) introduced the UCB-MSCs injection under ultrasound guidance to rabbits with acute full-thickness subscapularis tendon tears and revealed that UCB-MSCs promoted the partial regeneration of rotator cuff tendon tears with improved histologic appearance, tendon size, and walking capacity. Then, they investigated the efficacy of UCB-MSCs for chronic full-thickness rotator cuff tendon tears without repair and found that the injection of UCB-MSCs had a similar therapeutic effect in histological examination and motion analysis of walking 4 weeks after treatment (Rak Kwon et al., 2020). However, there was no difference between the high-dose and low-dose (2 × 10^6^ and 1 × 10^6^ cells, respectively) of UCB-MSCs, indicating that the benefits of UCB-MSCs were not in a dose-dependent manner (Kwon et al., 2019). In another study, UCB-MSCs–seeded biomimetic hydroxyapatite-gradient scaffold regenerated the tendon–bone interface of the rotator cuff in a rat repair model in terms of improving collagen organization, cartilage formation, and similar biomechanical properties as the normal tendon–bone interface at 8 weeks (Yea et al., 2020).

#### 3.1.5 Bursa-Derived Cells

Previous studies have suggested that the subacromial bursa is an important source of pluripotent stem cell potency for tendon healing (Utsunomiya et al., 2013; Baldino et al., 2020). Bursa-derived stem cells (B-MSCs) are easily accessible stem cells that can be harvested from routine rotator cuff repair surgery (Baldino et al., 2020). Like other stem cells, B-MSCs demonstrate high proliferation ability and multipotential differentiation *in vitro* (Utsunomiya et al., 2013). Recently, Muench et al. (2020) proved that B-MSCs consistently exhibited high cellular proliferation regardless of patient demographics (age, sex, body mass index, smoking status, and presence of systemic comorbidities), characteristics of rotator cuff tear (size, tendon retraction, fatty infiltration, and muscle atrophy), and the severity of glenohumeral joint degeneration.

Another study found that B-MSCs isolated from human bursae were characterized by multilineage differentiation, including osteoblastic, adipogenic, chondrogenic, and tenogenic lineages *in vitro* and *in vivo*. Additionally, they were induced into the bone, fibrocartilage, and tendon under different environments or pretreatments (Song et al., 2014). Compared with B-MSCs, cells isolated from bursae displayed superior engraftment and survival in tendon tissue and increased the thickness of the healing tissue compared with tissue that did not receive cells (Dyrna et al., 2018). Thus, it is suggested that B-MSCs are potent promising cells in rotator cuff injury; further studies should confirm their therapeutic effect for rotator cuff injury in pre-clinical and clinical studies.

#### 3.1.6 Urine-Derived Stem Cells

Currently, studies pay more attention to stem cells isolated from urine (USCs) due to their robust proliferation ability and multipotential differentiation into osteocytes, chondrocytes, adipocytes, neurocytes, and myocytes (Bharadwaj et al., 2013; Ji et al., 2017). The obvious advantage of USCs is that the harvest method is noninvasive and accessible. As autologous stem cells, USCs exhibit low immunogenicity, which may cause a low rejection response during treatment. Therefore, USCs are considered an attractive source of stem cells for rotator cuff healing. Chen Y. et al. (2020) implanted an autogenous, TGF-β3-induced USC sheet to the injured site of rotator cuff repair, evident by increased bone volume and trabecular thickness, which yielded enthesis-like tissue with more proteoglycan and collagen, as well as higher failure load and stiffness in comparison to the control group only at 12 weeks post repair. Nevertheless, more studies are required to evaluate the efficacy of untreated USCs on both acute and chronic rotator cuff injuries to offer a better research basis for future clinical transplantation.

### 3.2 Extracellular Vesicle-Based Therapy

MSCs are thought to mediate therapeutic functions in a paracrine manner in addition to their multipotent differentiation capacity and direct intercellular interactions. In the example of a rotator cuff injury model, conditioned medium (CM) of human bone marrow-derived stem cells promotes rotator cuff healing by increasing histologic score, bone mineral density and biomechanical tensile after surgery (Reiner et al., 2017; He et al., 2021). To search for the therapeutically active components, Bruno and colleagues successfully fractioned CM of MSCs by ultracentrifugation and discovered the therapeutic vesicular structures (Bruno et al., 2009). Collectively, these nano-sized particles with a lipid bilayer, naturally released by cells, are called extracellular vesicles (EVs) (Théry et al., 2018). They can be categorized into exosomes, microvesicles, and apoptotic bodies according to their cellular origins. Exosomes are the smallest vesicle types (40–120 nm), and they originate from the inward budding of late multivesicular endosomes (MVEs) and are released upon fusion of MVEs with the plasma membrane. Meanwhile, microvesicles are formed by budding from the plasma membrane, and their size can vary from 50 nm to 1 μm. Unlike exosomes and microvesicles, which are released by all cells, apoptotic bodies are vesicles (50 nm–5 μm) produced by cells undergoing apoptosis. EVs have become an attractive approach in regenerative medicine because they exert biological activities like those of stem cells and overcome the shortages of cell-based therapy, such as cell expansion, low survival rate, and potential immunological rejection (Keshtkar et al., 2018; Woo et al., 2020). It is believed that MSCs cultured in chemically defined serum-free media may be more suitable for the manufacture of EVs. The methods of separation and concentration may vary depending on the size of EVs and the purpose of end-use. According to a worldwide ISEV survey in 2015, ultracentrifugation was the most widely used primary EV separation and concentration technique (Gardiner et al., 2016). A variety of techniques have also been developed to achieve better specificity of separation, such as density gradients and size-exclusion chromatography (Théry et al., 2018). However, there are currently no accepted metrics for assessing the purity or degree of purity in EV preparation (Reiner et al., 2017). Although the mechanism of EVs is not fully understood, it is believed that EVs secreted by stem cells can promote tissue repair and regeneration by inducing the proliferation of cells, promoting angiogenesis, modulating the inflammation process, and affecting cell apoptosis (He et al., 2021). The therapeutic effects of MSC-EVs for rotator cuff injuries are summarized in Figure 2 and Table 3.

**TABLE 3:** Summary of extracellular vesicles from mesenchymal stem cells for the repair of rotary cuff injuries.

	Animal	Injury model	Source of EVs/extraction/dose/frequency	Delivery method/site	Time of observation	Results
Wang C. et al., (2020)	Rabbit	Acute tear and repair of supraspinatus tendon	human ADSC-derived EVs/ultracentrifugation/1 ×10^11^ particles/once	Saline/distal site of the supraspinatusmuscle	6 and 18 weeks	ASC-Evs showed significantly lower fatty infiltration, a higher histological score and more newly regenerated fibrocartilage at the repair site and biomechanical properties than the saline group
Huang et al., (2020)	Rat	Chronic tear and repair of supraspinatus tendon	Rat BMSC-derived EVs/ultracentrifugation/200 μg/once	PBS/tail vein	4 and 8 weeks	BMSC-Evs increased the breaking load and stiffness of the rotator cuff after repair, induced angiogenesis around the rotator cuff endpoint, and promoted growth of the tendon–bone interface
Wang et al., (2021)	Mice	Chronic supraspinatus tendinopathy	Human ADSC-derived EVs/ultracentrifugation/1 ×10^11^ particles/once	Saline/enthesis site of the supraspinatusmuscle	4 weeks	Mice in the ASC-Evs group showed less cellular infiltration, disorganization of collagen, and ground substance deposition, and higher maximum failure load and stiffness, than that of the saline group
Fu et al., (2021)	Rat	Chronic tear and repair of supraspinatus tendon	Human ADSC-derived EVs/ultracentrifugation/300 μg/once	Commercial hydrogel/shoulder site (no detailed information)	4 and 8 weeks	The ADSC-EVs have less inflammation and mire regular alignment and greater the expression of *RUNX2*, *Sox9*, *TNMD*, *TNC* and Scx, and higher mechanical properties compared with other groups
Kim et al., (2022)	Rabbit	Chronic tear and repair of supraspinatus tendon	Human umbilical cord-derived EVs/ultracentrifugation/2.9 ×10^9^ particles/once	Collagen/bursal side of the repaired site	12 weeks	The umbilical cord-derived EVs showed greater histomorphometric total score of collagen maturation in bone–tendon interface, lower fatty degeneration, and growing trends in mechanical properties as compared with applying collagen only or repair only

Notably, the most important impact of EVs on tissue regeneration is their immunomodulatory properties at both humoral and cellular levels. They can reduce injury-induced inflammation by dampening but not inhibiting complement activation through CD59, and they involve the promotion of anti-inflammatory and pro-regenerative (M2) macrophages over pro-inflammatory M1 macrophages and concomitantly enhance the expression of anti-inflammatory cytokines such as IL-10 instead of pro-inflammatory cytokines such as IL-1β and TNF-α (Toh et al., 2018). Additionally, EVs contain a large amount of biological information, including biologically mRNAs, miRNAs, and lncRNAs, which are important for modulating the signaling of the endogenous and exogenous cells of the injured site (Forsberg et al., 2020). Because of this advantage, engineered EVs are also regarded as candidate cargo to realize gene therapy for injuries. Additionally, the different resources of human MSC-derived EVs can exhibit distinct characteristics that reveal their potential applications in different fields. Bone marrow MSC-derived EVs have shown superior regeneration ability, and adipose tissue MSC-derived EVs have played a significant role in immune regulation, whereas umbilical cord MSC-derived EVs are prominent in tissue damage repair (He et al., 2021).

BMSC-derived EVs (BMSC-EVs) are widely used in the musculoskeletal regeneration field. Recently, one study solely injected BMSC-EVs into the vein to promote rotator cuff repair (Huang et al., 2020). The results showed that BMSC-EVs promoted angiogenesis around the tendon–bone interface, histologic histological appearance, and biomechanical strength, and they inhibited the secretion of pro-inflammatory factors in rat rotator cuff reconstruction. They believed that the mechanism by which BMSC-EVs achieve the healing process may be through the proliferation, migration, and angiogenic tube formation of human umbilical vein endothelial cells (HUVECs) by regulating the angiogenic signaling pathway, inhibiting the polarization of M1 macrophages, and also inhibiting the secretion of pro-inflammatory factors by M1 macrophages (Huang et al., 2020). A previous study reported BMSC-EVs can also suppress inflammation by increasing the expression of anti-inflammatory mediators IL-10 and IL-4 at an early phase of healing (Shi et al., 2019). Moreover, local delivery of BMSC-EVs can promote tendon regeneration by facilitating the proliferation, migration, and tenogenesis differentiation of endogenous TPSCs (Shi et al., 2019; Yu et al., 2020).

ADSC-derived EVs (ADSC-EVs) have regeneration and immunomodulation capacities (Chen et al., 2021; Wang et al., 2021). To investigate the effect of ADSC-EVs on chronic rotator cuff tears, Wang C. et al. (2020) established a bilateral rotator cuff chronic tear model and injected isolated ADSC-EVs at the injury site of the supraspinatus muscle at the time of repair. This demonstrated that the local injection of ADSC-EVs inhibited fatty infiltration, regenerated fibrocartilage, and increased biomechanical strength. Similarly, Fu et al. (2021) reported that rotator cuff injected with ADSC-EV-loaded hydrogel exhibited aligned collagen fiber and muscle bundles and enhanced mechanical properties. A variety of mechanisms may contribute to ADSC-EVs in rotator cuff repair. TPSCs regulate the proliferation, migration, and tenogenic differentiation of TPSCs. Studies have reported that ADSC-EVs can upregulate the expression of the tenogenesis genes *TNMD*, *TNC*, and *Scx in vivo* (Liu H. et al., 2021; Fu et al., 2021). Smad signaling pathways play vital roles in regulating stem cell activity. This effect may be related to activated SMAD2/3 and SMAD1/5/9 signaling pathways, which play vital roles in regulating stem cell activity (Liu H. et al., 2021). In addition, ADSC-EVs regulate the early inflammatory response in rotator cuff healing by decreasing the M1 macrophage, enhancing the M2 macrophage, and reducing the secretion of pro-inflammatory cytokines, such as IL-1β, IL-6, IL-8, and MMP-9 (Liu H. et al., 2021). In a human supraspinatus explant experiment, ADSC-EVs maintained homeostasis of the impaired tendon by increasing expression of *COL1A1*, *COL3A1*, and an elevated type I/III ratio and by decreasing expression of *MMP-9* and *MMP-13* (Zhang et al., 2021). The underlying mechanism might be enhancing AMPK signaling to suppress Wnt/β-catenin activity or NF-κB pathway (Ma et al., 2019; Zhang et al., 2021).

Few research observations of TPSC-derived EVs (TPSC-EVs) exist on rotator cuff repair. Recently, a study reported that TPSC-EVs suppressed inflammation and apoptosis at 1 week after surgery; the tendon exhibited a more continuous and regular arrangement and a larger collagen fiber diameter in the TPSC-EV-treated group compared to the non-TPSC-EV-treated group at two and eight weeks after surgery (Zhang et al., 2020a). The augment of TPSC-EVs can be partially explained by promoting the proliferation and migration of tenocytes in a dose-dependent manner in an *in vitro* study, which was related to the activation of the PI3K/AKT and MAPK/ERK1/2 signaling pathways (Zhang et al., 2020a). Nevertheless, the detailed mechanism of TPSC-EVs is still poorly understood and needs further investigation.

Many studies have reported the potential of human umbilical cord mesenchymal stem cell-derived EVs (HUMSC-EVs) in tendon repair. Kim et al. (2022) demonstrated that the HUMSC-EVs laden injectable collagen could effectively promote bone-to-tendon healing *via* collagen maturation in the bone–tendon interface and prevent fatty degeneration of the rotator muscle at 4 weeks after rotator cuff repair. In a rat Achilles tendon injury model, treatment with HUMSC-EVs improved the histological structure, enhanced tendon-specific matrix components, and optimized biomechanical properties of the Achilles tendon, which was related to the overexpression of miR-29a-3p regulated by PTEN/mTOR/TGF-β1 signaling (Yao et al., 2021).

### 3.3 Gene Therapy

An increasing number of studies have utilized gene therapy to enhance and expand the therapeutic effectiveness of stem cells in tendon repair. In comparison to conventional stem cell therapy, modified stem cells or their EVs yield more production of a gene in the local injured site, with greater biological activity and lower immune response. There are two main methods of transferring the gene to target cells: viral and non-viral vector methods. Viral vectors have been widely used in gene therapy due to their high efficiency in gene delivery into the cells they infect. Commonly, recombinant viruses include adenovirus, lentivirus, retrovirus, and adeno-associated virus in tendon repair. However, the use of viral vectors may also meet the challenges of high-cost expenses and safety issues. Thus, non-viral vectors, such as plasmids, increase the interest of researchers in gene delivery because of their safety, simple manufacture, and lower immunogenicity.

Concerning rotator cuff regeneration, many studies have focused on facilitating the tenogenic differentiation of stem cells to promote rotator cuff repair. Gulotta et al. (2011a) showed that BMSCs transfected to overexpress *Scx* promoted the formation of fibrocartilage at the tendon insertion and improved biomechanical strength at 4 weeks for rats who underwent unilateral detachment and repair of the supraspinatus tendon. Owing to the tear of enthesis (Zones 3 and 4) in rotator cuff injuries, it was meaningful to transfer osteogenic and chondrogenic genes to enhance tendon–bone healing. It was demonstrated that, when transfected to overexpress developmental genes, membrane type 1 matrix metalloproteinase (MT1-MMP), which is thought to direct the process of ossification, promotes the formation of fibrocartilage at the tendon insertion and improves biomechanical strength (Gulotta et al., 2010). However, ADSC transduced with the osteogenic factor bone morphogenetic protein 2 (BMP-2) led to impaired healing by losing bone mass and decreasing biomechanical properties (Lipner et al., 2015).

In terms of chondrogenic genes, the genetically modified BMSCs with overexpressing BMP-13 showed no improvement in either biomechanical parameters or histological appearance in acute rotator cuff repair (Gulotta et al., 2011b). Additionally, gene-modified stem cells can inhibit inflammation during the healing process. Cheng et al. (2014) discovered that the silent TNF-α stimulated gene/protein 6 (TSG-6) of TPSCs reduced biomechanical strength, indicating that TPSCs might promote rotator cuff healing through regulating anti-inflammatory response by TSG-6 signaling. Schnabel et al. (2009) explored using the insulin-like growth factor-I (IGF-1) gene enhanced BMSCs significantly improved tendon histological scores and reduced ECM degradation in collagenase-induced bilateral tendinitis lesions, but the benefit of IGF-1 gene enhancement was not obvious compared to untreated BMSCs.

EVs contain miRNAs from donor cells that can be transferred to recipient cells, thereby promoting the expression of specific proteins. Therefore, an increasing number of studies pay attention to utilizing engineered EVs to transfer genes in musculoskeletal disorders such as osteoporosis (Yang et al., 2020) and osteoarthritis (Tao et al., 2017). Several studies have demonstrated the promising results of engineered EVs in tendon repair and regeneration. Early tendon remodeling plays a vital role in tendon regeneration. Yao et al. (2021) revealed that miRNA-29a-3p loaded HUMSC-EVs reduced the area of the lesion and improved histological scores in a tendinopathy model. The underlying pathway was reducing transcript levels of collagen III *via* the PTEN/mTOR/TGF-β1 signaling pathway (Millar et al., 2015; Watts et al., 2017).

Another strategy of gene therapy for tendon healing is to inhibit the fibrous process of the tendon and surrounding tissues. The EVs derived from antagonists targeting miR-21a-3p treatment of HUMSC, which expressed low levels of miR-21a-3p, expanded the inhibition of tendon adhesion by manipulating p65 activity, suggesting that delivering low-abundance miR-21a-3p may inhibit tendon adhesion. Furthermore, some miRNAs participate in tenogenic differentiation and prevent chondro-osteogenic differentiation, including miR-124, miR-135a, miR-140, and miR-337-3p (Chen et al., 2015; Wang et al., 2016; Geng et al., 2020; Liu Y. J. et al., 2021). However, the effectiveness of the delivery of these genes by EVs has not been confirmed.

## 4 Biomaterials

Innovation in the field of biomaterials has driven the development of regenerative medicine and tissue engineering. Biomaterials are bioresorbable and gradually degraded so that tissues have sufficient space for regeneration as well as negligible immunogenicity and side effects locally and systematically (Garg et al., 2012). In rotator cuff repair, biomaterials used for stem cell or EV delivery can be divided into two categories: implantable and injectable delivery systems (Chen et al., 2019; Liu et al., 2020). Commonly, an implantable delivery system is composed of tissue engineering scaffold biomaterials, which should have a three-dimensional structure that allows for cell attachment, growth, and proliferation. In addition, biomaterials used for injectable delivery systems are supposed to carry bioactive factors and cells to the target injury site while minimizing the spread of drugs. Injectable deliveries have the advantage of a minimally invasive nature, but they cannot provide sufficient support for cells and impaired tissues. The delivery system in rotator cuff repair includes, decellularized tissues, electrospun nanofiber scaffolds, hydrogels, and patterned scaffolds, but is not limited to these (Longo et al., 2012; Saveh-Shemshaki, 2019).

### 4.1 Biomaterial Polymers

To date, various natural and synthetic materials have been developed to promote stem cells in rotator cuff repair and regeneration. In our review, natural polymers are most widely used in stem cell therapies for rotator cuff injuries (Gulotta et al., 2010; Gulotta et al., 2011a; Degen et al., 2016; Liu et al., 2019). They have the advantages of non-toxicity, biocompatibility, and biodegradation, as well as cell proliferation and cell adhesion. Nevertheless, it is difficult to modify their physical and chemical properties, which remains a potential immunogenicity problem (Garg et al., 2012). Commonly used natural materials include ECM-derived biomaterials, hyaluronic acid (HA), chondroitin sulfate (CS), and fibrin. As the main component of the ECM, ECM-based biomaterials provide a biomimetic environment suited for tissue remodeling. For instance, Liu et al. (2019) reported a novel biomaterial that uses engineered tendon–fibrocartilage–bone composite (TFBC) augmentation with BMSCs to form a “sandwich” structure that can enhance rotator cuff healing in terms of anatomic structure, collagen organization, and biomechanical strength. Decellularized matrices have been explored for their regenerative effects on tendon repair; however, tissue resources should be considered. While the tendon-derived decellularized matrix promoted the tendinous phenotype in TSPCs and inhibited their osteogenesis, the dermal skin-derived collagen matrix had no apparent effect on TSPC differentiation (Yin et al., 2013). Hyaluronic acid (HA) is an anionic, non-sulfated glycosaminoglycan that is distributed in the intercellular matrix of most connective tissues. It has been reported that HA decreases the cell proliferation and expression level of procollagen α1 (III) mRNA of tendon-derived fibroblasts (Yamada et al., 2007). Moreover, CS is a natural polymer and a major ECM component that has the ability to reduce inflammation by diminishing NF-κB activation and nuclear translocation (Vallières and Du Souich, 2010). Human mesenchymal stem cells cultured within the decellularized amniotic matrix wrapped around the collagen-chondroitin sulfate scaffold could maintain metabolic activity and down-regulate the pro-inflammatory cytokines (Hortensius et al., 2018). Fibrin is formed following the cleavage of fibrinogen and thrombin and can be processed into hydrogels or fibrous scaffolds. Tissue-engineered construction based on fibrin hydrogel has better extracellular matrix organization and biomechanical properties compared to collagen-based hydrogels (Breidenbach et al., 2015; Thangarajah et al., 2018).

Synthetic materials are also used extensively in tendon regeneration since their molecular weight, hydrophobicity, and degradation speed can be easily modified, and due to their low cost of fabrication (Ruiz-Alonso et al., 2021). Notably, it is possible to achieve similar mechanical properties with tendon tissue and good structural integrity, which are important in the regeneration of tendon repair (Pina et al., 2019). Compared to natural biomaterials, synthetic materials have a low risk of disease transmission because they are not obtained from biological organisms or tissues. For this reason, they have a high risk of immune response (Won et al., 2014). Since synthetic materials are hydrophobic in nature, they may also cause poor cell adherence, low proliferation rates, and altered phenotypes of stem cells (Theisen et al., 2010). Numerous synthetic materials are used for tendon tissue repairs, such as poly-ε-caprolactone (PCL), poly (lactic acid) (PLA), poly (glycolic acid) (PGA), poly (ethylene glycol) (PEG), and poly (lactic-co-glycolic acid) (PLGA). These synthetic polymers can be electrospun into nano- and microfibrous scaffolds, which mimic aligned collagen fibers in tendon tissue and promote tenogenic differentiation (Vuornos et al., 2016; Laranjeira et al., 2017; Calejo et al., 2019; El Khatib et al., 2020). The rate of degradation determines its usage. For example, polymers with a low degradation rate, such as PCL, are suitable for building longer-term tendon scaffolds (Laranjeira et al., 2017; Calejo et al., 2019), while polymers with faster degradation rates are less suitable since they may increase the inflammation response, including PLA, PGA, and PLGA (Yokoya et al., 2008; Vuornos et al., 2016; Chen et al., 2019; Chen P. et al., 2020; Araque-Monrós et al., 2020; El Khatib et al., 2020). According to this characteristic, a PEG-based hydrogel system with a range of degradation rates can control the timing of MSC delivery to the target site of tendinopathy (Qiu et al., 2011).

### 4.2 Interactions Between Biomaterials and Stem Cells

The topographical and mechanical properties of biomaterials impact the proliferation and tenogenic differentiation of stem cells, including fiber diameter, pore size, alignment, surface roughness, and matrix stiffness. Fiber with a large diameter promotes the expression of tenogenic genes, such as *Scx*, in stem cells. This suggests that large-diameter fibers (e.g., >2 μm) may be more suitable for MSC differentiation into tendon lineage than small-diameter fibers (Cardwell et al., 2014). Generally, the pore size of the scaffold plays an important role in migration ability (Zheng et al., 2017); a larger pore size of PLGA scaffolds significantly enhances the migration of BMSCs *in vitro* (Dai et al., 2018). Fiber alignment provides tissue-specific biomechanical cues to resident cells in the native tendon. Studies have indicated that scaffolds with aligned fibers enhance cell infiltration, ECM deposition, collagen alignment, and tendon-related gene expression of stem cells when compared to nonaligned fibers (Orr et al., 2015; Zheng et al., 2017). In the massive rotator cuff repair model, scaffolds with aligned fibers exhibit more conspicuous native microstructures, better alignment, and better mechanical properties at 12 weeks post-implantation (Zheng et al., 2017). Due to the mechanical microenvironment of the tendon, matrix stiffness impacts stem cells during tendon repair. The proliferation of TPSCs increases and more stress fibers form with increasing matrix stiffness. Furthermore, the differentiation of TPSCs into tenogenic lineages is inhibited on stiff hydrogel with reduced expression of tendon-specific genes *THBS4*, *TNMD*, and *SCX* by regulating FAK and ERK1/2 pathways (Liu et al., 2018).

Concerning rotator cuff repair, the scaffold is an effective tool for transmitting mechanical stimulation to delivered cells; thus, the mechanical environment provided by biomaterials should be considered in cell delivery. The mechanical stimulation of stem cells is vital in tendon tissue repair and has been shown to influence the differentiation and proliferation of stem cells (Wang H.-N. et al., 2020). The magnitude of stretching could lead to different cell fates. Studies indicated that 4% stretching promoted the differentiation of TPSCs into tenocytes with increased gene expression of *COL1A1*; 8% stretching, however, promoted the differentiation of TPSCs into non-tenocytes, including adipocytes, chondrocytes, and osteocytes, aside from differentiation into tenocytes, as evidenced by higher expression levels of genes such as *PPARγ*, *COL2A1*, *Sox9*, and *Runx2 in vitro* (Wang H.-N. et al., 2020).

Furthermore, mechanical stimulation of BMSCs significantly increased the expression of tenogenic genes and anti-inflammatory cytokines (Ciardulli et al., 2020). In addition, biomaterials containing magnetic elements have been developed to mechanically stimulate stem cells in tendon regeneration. Human adipose stem cells cultured on the magneto-mechanical actuation scaffold increased the expression of tendon-related genes *Scx* and *Tnmd* when compared to static culture and steered the mechanosensitive YAP/TAZ signaling pathway. This magneto-mechanical stimulation also modulated the inflammation response by upregulating the expression of anti-inflammatory cytokines IL-4 and IL-10 while reducing the expression of pro-inflammatory cytokines COX-2 and IL-6 (Tomás et al., 2019).

### 4.3 Responsive Biomaterial Strategies for Rotator Cuff Injury

To achieve the different requirements of delivery, “smart” delivery systems, such as stimulation-responsive hydrogel, can provide possibilities for precise treatment for different stages of healing (Bawa et al., 2009; Yun et al., 2015). These hydrogels respond to visible or UV light and release drugs for tissue regeneration. A gelatin methacryloyl hydrogel loaded with TPSC-EVs was placed in the Achilles tendon defect to promote tendon healing. By using a 405 nm blue light source at a distance, the carrier is converted to the gel state by irradiation for 10–20 s. After delivering TPSC-EVs, tendon repair is promoted by suppressing inflammation and apoptosis and regulating ECM balance (Zhang et al., 2020b). Additionally, matrix metalloproteinase 2 (MMP-2) is a valuable endogenous trigger for responsive release systems, achieving localized and on-demand drug delivery. MMP-2 is a member of the zinc endopeptidase family and has the ability to cleave ECM components, which is upregulated in the peritendinous area where the adhesion tissue forms after tendon injury. Cai et al. (2021) have designed an innovative anti-adhesion electrospun nanofiber scaffold system for the on-demand and unidirectional release of polyplexes to inhibit fibroblast proliferation and collagen deposition by gene therapy. The MMP-2 degradable hydrogel is fabricated by crosslinking allyl glycidyl ether (AGE) modified carboxymethyl chitosan (CMCS-AGE) and the MMP-2 substrate peptide CPLGLAGC (MMP-2 sp).

## 5 Discussion

Rotator cuff injuries cause persistent symptoms, and they greatly impair movement ability and quality of life. Currently, the clinical options of surgery and conventional therapies for treating rotator injuries are unsatisfactory. Due to the special structures of the rotator cuff, several clinical problems have not been solved, such as the delayed healing process, poor biomechanical strength of newly formed tissue, and scar adhesion. Therefore, stem cell therapies are attractive because they activate the self-potential of the body to repair injured tissues. According to present pre-clinical and clinical studies, several stem cells have been successfully isolated and have shown promising potential in rotator cuff repair due to their strong capacity for regeneration, tenogenic differentiation, and paracrine activity. As a primary effector in stem cell therapy, EVs can promote the healing process by reducing inflammation and fatty infiltration, stimulating cell proliferation and tenogenic differentiation, and maintaining homeostasis. Moreover, gene therapy and biomaterials expand the effectiveness of the application of stem cell therapy by regulating the environment, stimulating directional differentiation, and ensuring high efficacy of delivery. Therefore, stem cell therapy is a promising strategy for rotator cuff repair.

Nevertheless, numerous issues still need to be investigated in future studies. Although more stem cells and their EVs, such as Bursa-derived cells, have been successfully discovered and isolated, the lack of pre-clinical and clinical studies limits their further application. Moreover, with the deepening research of gene therapy, the efficient, safe, and targeted gene vector and therapeutic genes need to be addressed and verified in large animal models before beginning clinical trials. Innovation in biomaterials is evolving rapidly; thus, the translation of safe and valid carriers is the key to advancing the clinical application of stem cell therapy. Finally, to fully understand the safety, effectiveness, and mechanism of stem cell therapy, basic clinical research is still required.
